# Supplementary material for: Properties of a Novel Salmonella Phage L66 and Its Application Based on Electrochemical Sensor-Combined AuNPs to Detect Salmonella
Source: Foods. 2022 Sep 13;11(18):2836. doi: 10.3390/foods11182836 (PMC9498146; doi:10.3390/foods11182836)
Supplement: Supplementary file 1 [file foods-11-02836-s001.zip › foods-1893516-supplementary.pdf]

foods MDPI

Supplementary information

Properties of a Novel *Salmonella* Phage L66 and its Application Based on Electrochemical Sensor-Combined AuNPs to Detect *Salmonella*

Changbin Li [1,2], Yuanshang Wang [1], Jia Wang [1] and Xiaohong Wang [1,3,*]

[1] College of Food Science and Technology, Huazhong Agricultural University, Wuhan 430070, China
[2] College of Food and Biology Engineering, Henan University of Animal Husbandry and Economy, Zhengzhou 450046, China
[3] Key Laboratory of Environment Correlative Dietology, Huazhong Agricultural University, Wuhan 430070, China
* Correspondence: wxh@mail.hzau.edu.cn; Tel.: +86-027-8728-2111

Table S1. Host spectrum of phage L66.

Number	G^+/G^{-1}	Strain	Source	R/S	Species	Lytic ability
1	G^-	SJTUF 10978	LC	S	*S.* Enteritidis	+++
2	G^-	SJTUF 10984	LC	S	*S.* Enteritidis	++
3	G^-	38	LC	R	*S.* Enteritidis	+++
4	G^-	39	LC	R	*S.* Enteritidis	+++
5	G^-	42	LC	R	*S.* Enteritidis	+++
6	G^-	201	LC	R	*S.* Enteritidis	+
7	G^-	211	LC	R	*S.* Enteritidis	++
8	G^-	10960	LC	R	*S.* Enteritidis	++
9	G^-	11561	LC	R	*S.* Enteritidis	+++
10	G^-	ATCC 14028	ATCC	S	*S.* Typhimurium	+++
11	G^-	ATCC 13311	ATCC	S	*S.* Typhimurium	+
12	G^-	30	LC	R	*S.* Typhimurium	—
13	G^-	36	LC	R	*S.* Typhimurium	—
14	G^-	114	LC	R	*S.* Typhimurium	++
15	G^-	172	LC	R	*S.* Typhimurium	+
16	G^-	206	LC	R	*S.* Typhimurium	+++
17	G^-	10855	LC	R	*S.* Typhimurium	++
18	G^-	SJTUF 13306	LC	R	*S.* Typhimurium	++
19	G^-	SJTUF 13277	LC	R	*S.* Typhimurium	+
20	G^-	SJTUF 13336	LC	R	*S.* Typhimurium	++
21	G^-	SJTUF 13337	LC	R	*S.* Typhimurium	++
22	G^-	SJTUF 13350	LC	R	*S.* Typhimurium	++
23	G^-	UK-1	LC	S	*S.* Typhimurium	+++
24	G^-	NCTC 12900	NCTC	S	E. coli	—
25	G^-	ATCC 25923	ATCC	S	E. coli	—
26	G^-	12900	LC	S	E. coli	—
27	G^-	ATCC 29544	ATCC	S	E. coli	—
28	G^-	DH5α	LC	S	E. coli	—
29	G^-	CICC10662	CICC	S	E. coli	—
30	G^-	CICC10664	CICC	S	E. coli	—
31	G^-	CICC10667	CICC	S	E. coli	—
32	G^-	CICC10669	CICC	S	E. coli	—

33	G^-	17	LC	R	*S.* Agonistes	+
34	G^-	19	LC	R	*S.* Agonistes	+
35	G^-	21	LC	R	*S.* Agonistes	+
36	G^-	3710	LC	S	*S.* Dublin	++
37	G^-	3723	LC	S	*S.* Dublin	++
38	G^-	CVCC 534	CVCC	S	*S. Pullorum*	++
39	G^-	CVCC 519	CVCC	S	*S. Pullorum*	+++
40	G^-	13500	LC	R	*S. Indiana*	++
41	G^-	13520	LC	R	*S. Indiana*	++
42	G^-	ATCC 9270	ATCC	S	*S. Duck*	+
43	G^-	ATCC 10708	ATCC	S	*S. Choleraesuis*	—
44	G^+	ATCC 19114	ATCC	S	*Listeria* monocytogenes	—
45	G^-	33846	ATCC	S	*Vibrio parahaemolyticus*	—
46	G^+	260031	CMCC	S	*S.* aureus	—
47	G^+	25923	ATCC	S	*S.*aureus	—
48	G^+	ATCC 29213	ATCC	S	*S.* aureus	—
49	G^+	ATCC 6538	ATCC	S	*S.* aureus	—

Note: G^+ for Gram-positive bacteria, G^- for Gram-negative bacteria. R for Resistant strain, S for Sensitive strain. "++++, ++, +, -,"refer to the different ability to infest different host bacteriophages of phages. "++++" indicates very transparent and clear phage spot, "+++" indicates clear and transparent phage spot, "+" indicates a hazy background of phage spot, and "-" indicates no phage spot phenomenon. "LC"refer to Laboratory collection.
